# Ulcerative colitis associated with nephrotic syndrome after treatment with mesalazine developed into rectal carcinoma: a case study

**DOI:** 10.1186/s12957-016-0950-1

**Published:** 2016-07-22

**Authors:** Shinichi Sameshima, Shinichiro Koketsu, Takashi Okuyama, Yawara Kubota, Yuko Ono, Tamaki Noie, Masatoshi Oya

**Affiliations:** Department of Surgery, Dokkyo Medical University Koshigaya Hospital, 2-1-50, Minami Koshigaya, Koshigaya, Saitama 343-8555 Japan; Department of Pathology, Dokkyo Medical University Koshigaya Hospital, 2-1-50, Minami Koshigaya, Koshigaya, Saitama 343-8555 Japan

**Keywords:** Ulcerative colitis, Mesalazine, Dysplasia, Carcinoma, Nephrotic syndrome, Steroid, Immunosuppressant

## Abstract

**Background:**

Previous studies reported that nephrotic syndrome is associated with ulcerative colitis (UC) patients treated with mesalazine. Dysplasia associated with UC often develops into colorectal carcinoma.

**Case presentation:**

A 17-year-old man was referred to our hospital, complaining of diarrhea and bloody stool. Total colonoscopy (TC) was performed and total-type UC was diagnosed. After treatment with mesalazine for 5 years, a low-grade dysplasia (LGD) was detected in the rectum by histological analysis of a biopsy sample. One month later, he complained of dyspnea and edema. He was diagnosed with nephrotic syndrome and administered steroid and immunosuppressant treatment: cyclosporine and mizoribine. Eight years after LGD was detected, he complained of abdominal distension and pain. Stenosis of the upper rectum by an advanced rectal carcinoma was detected. Abdominal computed tomography showed a rectal tumor with multiple lymph node metastases. Transverse colostomy was performed surgically, followed by two cycles of modified FOLFOX6 and panitumumab. He safely underwent a total proctocolectomy with a stapled ileal pouch anal-canal anastomosis, total mesorectal and bilateral pelvic lymph node dissection, and temporary loop ileostomy. Metastases were observed in 25 lymph nodes microscopically. The pathological stage of rectal carcinoma was pT3N2bM1a. After one cycle of modified FOLFOX6 postoperatively, he was discharged from the hospital.

**Conclusions:**

A patient with UC associated with nephrotic syndrome was treated with mesalazine. LGD developed into an advanced rectal carcinoma after an 8-year interval. The use of immunosuppressants for the treatment of nephrotic syndrome might affect the development of rectal carcinoma.

**Trial registration:**

Trial registration: Case report registration #1626

## Background

Long-standing ulcerative colitis (UC) patients are often associated with colorectal dysplasia or colorectal carcinoma (CRC). Patients with UC have a 2.4-fold increased overall risk of CRC [1]. The cumulative probability of UC patients developing CRC is 2 % by 10 years, 8 % by 20 years, and 18 % by 30 years, according to a meta-analysis [2].

Riddell described two categories of dysplasia associated with UC, *indefinite* and *positive* dysplasia [3]. The indefinite category includes the subcategories: probably negative (probably inflammatory), unknown, and probably positive (probably dysplastic). The positive category includes low-grade dysplasia (LGD) and high-grade dysplasia (HGD). It is therefore important to conduct surveillance colonoscopy to detect dysplasia before CRC develops [4].

Previous studies reported that UC patients treated with mesalazine were associated with nephrotic syndrome (NS) [5]. The relationship between mesalazine and NS is unclear. Steroids and immunosuppressants used for the treatment of NS might accelerate the advancement of dysplasia to colitic carcinoma.

The standard surgical procedure for patients with UC is restorative proctocolectomy with construction of an ileal pouch. Colectomy for UC patients with advanced carcinoma requires sufficient lymph nodes resections.

## Case presentation

A 17-year-old man complained of diarrhea and bloody stools and was referred to our hospital in 2000. His family had no particular disease. He was diagnosed with total-type UC by total colonoscopy (TC). The patient had no primary sclerosing cholangitis. Mesalazine at 2250 mg/day was prescribed. In June 2005, he underwent TC. Immunohistochemical analysis of a rectum biopsy showed LGD with an overexpression of p53 protein. The following month he complained dyspnea and edema of the whole body and he gained 4 kg in weight. He was consulted by a nephrology physician. Laboratory data showed total protein of 4.5 g/dl, albumin of 1.4 g/dl, total cholesterol of 352 mg/dl, creatinine of 1.7 mg/dl, and blood urea nitrogen of 43 mg/dl in the serum. His urine showed proteinuria 3.6 g/day. He was diagnosed with NS according to the criteria. Biopsy of the kidney demonstrated a minimal change in NS histologically. Prednisolone at 35 mg/day was initiated. Cyclosporine at 250 mg/day and mizoribine at 150 mg/day were added because of steroid resistance. His symptoms disappeared after 3 months of medication.

Mesalazine had not been administered to the patient since 2008. Prednisolone was tapered to 12.5 mg. TC was performed in 2013 and a stenotic lesion was detected in the rectum. Biopsy of the rectum showed LGD histologically.

In July 2014, he complained of abdominal distension. Abdominal X-ray showed distension of the colon and an abdominal computed tomography (CT) scan showed a rectal tumor associated with swellings of multiple abdominal lymph nodes. He was admitted to our hospital.

**Fig. 1:**
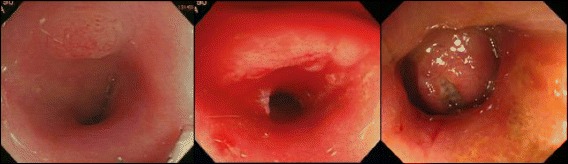
Endoscopic view of the rectal mucosa. Rectal mucosa with LGD in 2011 (*left*). Elevated and irregular rectal mucosa with LGD in 2013 (*center*). Rectal carcinoma in 2014 (*right*). *LGD* low-grade dysplasia

Laboratory data at admission is shown in Table 1. Serum carcinoembryonic antigen (CEA) and carbohydrate antigen 19-9 (CA19-9) levels were markedly elevated. Urinalysis did not show occult blood, proteinuria, and glycosuria. TC showed a round shaped elevated lesion in the rectum with an ulcer on top (Fig. 1). A colonoscope was passed through the stenosis site and inflammation of the colorectal mucosa was mildly active. Biopsy of the rectal tumor showed well-differentiated adenocarcinoma with mucin production. Overexpression of p53 protein was observed immunohistochemically. Magnetic resonance imaging (MRI) showed multiple swellings in the rectal mesentery lymph nodes and lateral pelvic lymph nodes.

**Table 1:** Laboratory data at admission

Hemoglobin	14.4 mg/dl
White blood cell	12,300/m^3^
Platelet	28.3 × 10^4^/m^3^
CEA	11.8 ng/ml
CA19-9	50.4 U/ml
AST	12 U/l
ALT	13 U/l
Total bilirubin	0.85 mg/dl
BUN	21.0 mg/dl
Creatinine	0.6 mg/dl
Total protein	6.1 g/dl
Albumin	3.94 g/dl
CRP	0.19 mg/dl

*CEA* carcinoembryonic antigen, *CA19-9* carbohydrate antigen 19-9, *AST* aspartate transaminase, *ALT* alanine aminotransferase, *BUN* blood urea nitrogen, *CRP* C-reactive protein

Transverse colostomy was performed under general anesthesia. As KRAS gene mutation was not detected in the biopsy specimen, the patient received a total of two cycles of modified FOLFOX6 and panitumumab [6] over 2 months as neoadjuvant chemotherapy. Adverse effects of chemotherapy were peripheral neuropathy (grade 1) and skin rash in the face (grade 1). Repeated CT scan and MRI did not show shrinkage of the tumor indicating stable disease. Five weeks after the completion of chemotherapy, the patient underwent a total proctocolectomy with a stapled ileal pouch anal-canal anastomosis, total mesorectal and bilateral pelvic lymph node dissection, and temporary loop ileostomy. The patient fared well in the postoperative course.

**Fig. 2:**
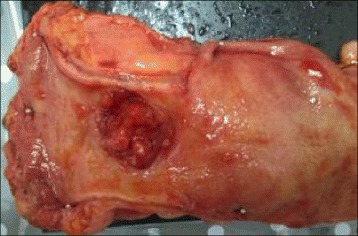
Macroscopic view of the resected specimen. UC with rectal carcinoma

**Fig. 3:**
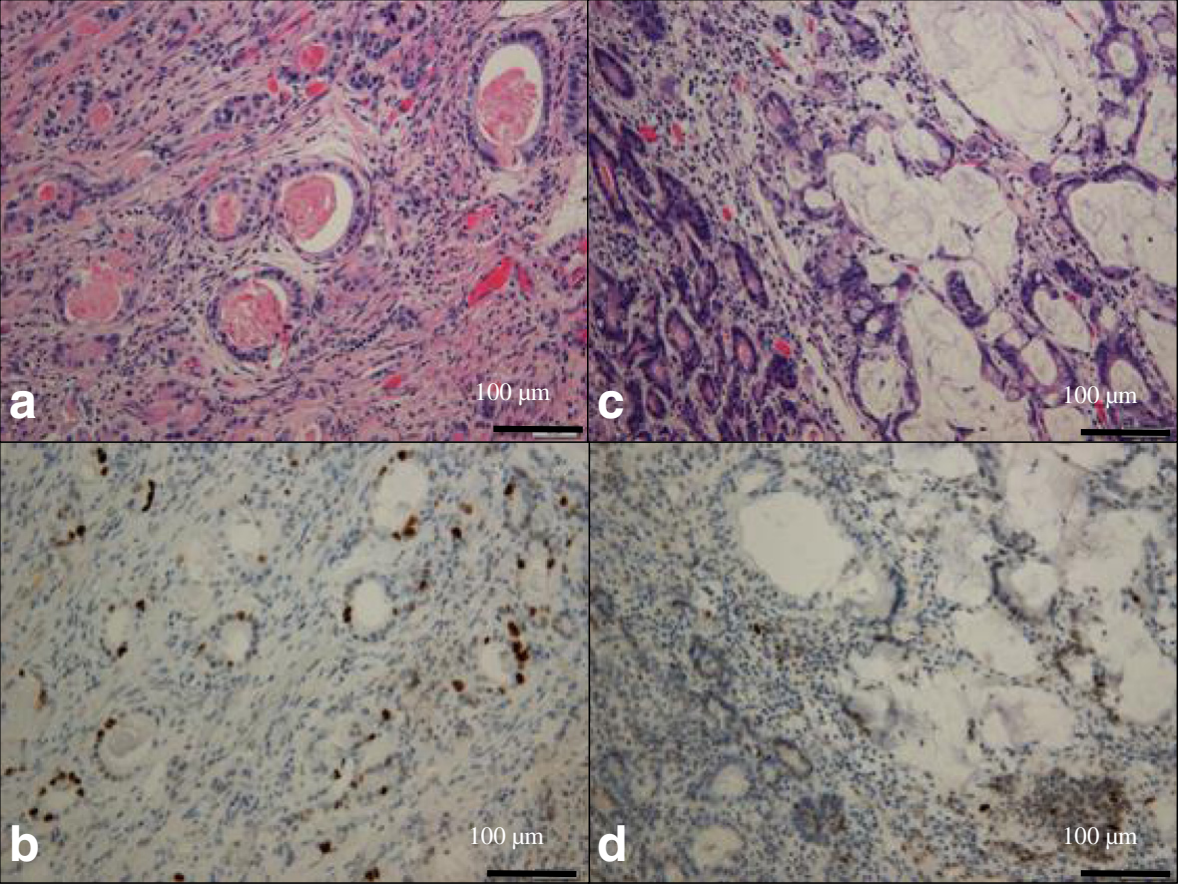
Middle power microphotograph of resected rectal carcinoma. Well-differentiated adenocarcinoma (**a**) and p53-positive staining are shown immunohistochemically (**b**). Moderately differentiated adenocarcinoma with mucinous carcinoma (**c**) and p53-positive staining are shown immunohistochemically (**d**)

The resected specimen showed an irregular shaped, round, elevated mass that was 3 cm in diameter at the rectum (Fig. 2). Background colorectal mucosa was flat with mild inflammation confirming UC. Histological examination of the specimen revealed mucinous adenocarcinoma with well and moderately differentiated adenocarcinoma. Over expression of p53 protein was observed immunohistochemically (Fig. 3). Metastases were observed in 25 lymph nodes including the mesorectal and bilateral pelvic lymph nodes. Colorectal mucosa showed active UC with cryptitis and crypt abscess histologically. The pathological stage of the carcinoma was pT3N2bM1a, stage IV, according to the TNM classification of UICC, 7th edition [7].

The patient was discharged from the hospital after receiving 1 cycle of modified FOLFOX6. He received additional 7 cycles of modified FOLFOX6 in 4 months at the outpatient unit. Adverse effects of postoperative chemotherapy were peripheral neuropathy (grade 2). The patient is currently alive without evidence of recurrence of the carcinoma 12 months after colectomy.

We report a case of an UC patient where disease was associated with advanced rectal carcinoma. UC was associated with NS after treatment of UC with mesalazine for 5 years. Firwana et al. reported a case of NS after mesalazine treatment for Crohn’s disease [5]. He also reviewed six case reports of NS after the treatment of UC with 5-amino-salacylic acid derivatives. All six patients were given steroids and improved. However, the relationship between mesalazine and NS is currently unclear.

Kiran et al. reported that carcinoma was present in 29 % of UC patients with preoperative HGD compared with 3 % in those with LGD [8]. Thus, the risk of carcinoma in UC patients with HGD is substantial. In the current case, the patient showed advanced rectal carcinoma 8 years after LGD was detected by surveillance colonoscopy. He received steroids and immunosuppressants for the treatment of NS. However, these reagents might accelerate the progression of rectal carcinoma from dysplasia.

The standard surgical procedure for patients with UC is restorative proctocolectomy with construction of an ileal pouch. Colectomy for UC patients with carcinoma requires sufficient lymph nodes resections [9]. In this case, 25 metastatic lymph nodes including the pelvic lateral lymph nodes were resected surgically. It is very rare that so many metastatic lymph nodes were detected in resectable sporadic or colitic carcinoma cases without distal organ metastases. This patient was under immunosuppressive conditions induced by the steroid and immunosuppressant treatment for NS. The possibility exists that this immunosuppressive condition was precipitated the aggressive lymph node metastases.

Advanced carcinoma affects the lifespan of UC patients. In a study conducted in Japan, Watanabe et al reported that patients with CRC-associated UC showed poorer survival rates than those with sporadic CRC in the advanced stages, while no difference was observed in the early stages [10]. It is important to detect dysplasia and carcinoma at an early stage by surveillance. LGD with an overexpression of p53 protein was detected in this patient by surveillance colonoscopy. p53 mutations have been shown to occur at an earlier phase in the progression of UC-associated neoplasia [11]. It had already developed to an advanced carcinoma when he underwent the follow-up colonoscopy. This is a rare case, and there are still problems concerning the method of surveillance and follow-up colonoscopy.

## Conclusions

A patient with UC treated with mesalazine developed associated NS. Although similar cases have been reported, a relationship between mesalazine and NS is not evident. In this case, LGD detected by surveillance colonoscopy developed to an advanced rectal carcinoma after an 8-year interval. The use of immunosuppressants for the treatment of NS might affect the progression of rectal carcinoma and aggressive lymph node metastases.

## Abbreviations

CA19-9, carbohydrate antigen 19-9; CEA, carcinoembryonic antigen; CRC, colorectal carcinoma; CT, computed tomography; HGD, high-grade dysplasia; LGD, low-grade dysplasia; MRI, magnetic resonance imaging; NS, nephrotic syndrome; TC, total colonoscopy; UC, ulcerative colitis
